# Direct isolation and characterization of circulating exosomes from biological samples using magnetic nanowires

**DOI:** 10.1186/s12951-018-0433-3

**Published:** 2019-01-07

**Authors:** Jiyun Lim, Mihye Choi, HyungJae Lee, Young-Ho Kim, Ji-Youn Han, Eun Sook Lee, Youngnam Cho

**Affiliations:** 1 Biomarker Branch, National Cancer Center, 323 Ilsan-ro, Ilsan-dong-gu, Goyang, Gyeonggi 10408 South Korea; 2 Department of Cancer Biomedical Science, Graduate School of Cancer Science and Policy, 323 Ilsan-ro, Ilsan-dong-gu, Goyang, Gyeonggi 10408 South Korea; 3 Department of Medical Science, Yonsei University College of Medicine, 50 Yonsei-Ro, Seodaemun-Gu, Seoul, 03722 South Korea; 4 Division of Clinical Research, Rare Cancer Branch, National Cancer Center, 323 Ilsan-ro, Ilsan-dong-gu, Goyang, Gyeonggi 10408 South Korea; 5 Division of Lung Cancer, National Cancer Center, 323 Ilsan-ro, Ilsan-dong-gu, Goyang, Gyeonggi 10408 South Korea; 6 Division of Breast Cancer, National Cancer Center, 323 Ilsan-ro, Ilsan-dong-gu, Goyang, Gyeonggi 10408 South Korea; 7 Genopsy Inc., 373 Kangnamdaero, Seocho-Gu, Seoul, 06621 South Korea

**Keywords:** Plasma, Exosome, Magnetic nanowire, Lung cancer, Breast cancer

## Abstract

**Background:**

Tumor-derived exosomes are gaining attention as important factors that facilitate communication between neighboring cells and manipulate cellular processes associated with cancer development or progression. The conventional techniques for the isolation and detection of exosomes face several limitations, restricting their clinical applications. Hence, a highly efficient technique for the isolation and identification of exosomes from biological samples may provide critical information about exosomes as biomarkers and improve our understanding of their unique role in cancer research. Here, we describe the use of antibody cocktail-conjugated magnetic nanowires to isolate exosomes from plasma of breast and lung cancer patients.

**Methods:**

The isolated exosomes were characterized based on size and concentration using nanoparticle tracking analysis. Levels of exosomal proteins were measured by bicinchoninic acid assay and enzyme-linked immunosorbent assay. Morphology was visualized by transmission electron microscopy. Immunoblotting (Western blotting) was used to detect the presence of exosomal markers.

**Results:**

The use of antibody cocktail-conjugated magnetic nanowires resulted in approximately threefold greater yield when compared to the conventional methods. The elongated feature of nanowires significantly improved the efficiency of exosome isolation, suggesting its potential to be translated in diverse clinical applications, including cancer diagnosis and treatment.

**Conclusions:**

The nanowire-based method allows rapid isolation of homogeneous population of exosomes with relatively high yield and purity from even small amounts of sample. These results suggest that this method has the potential for clinical applications requiring highly purified exosomes for the analysis of protein, lipid, mRNA, and miRNA.
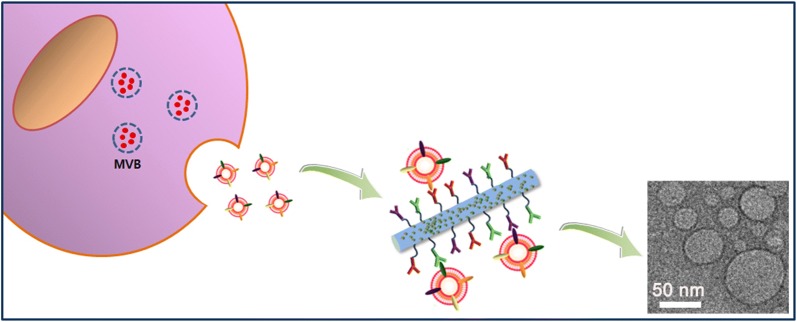

## Background

**Fig. 1:**
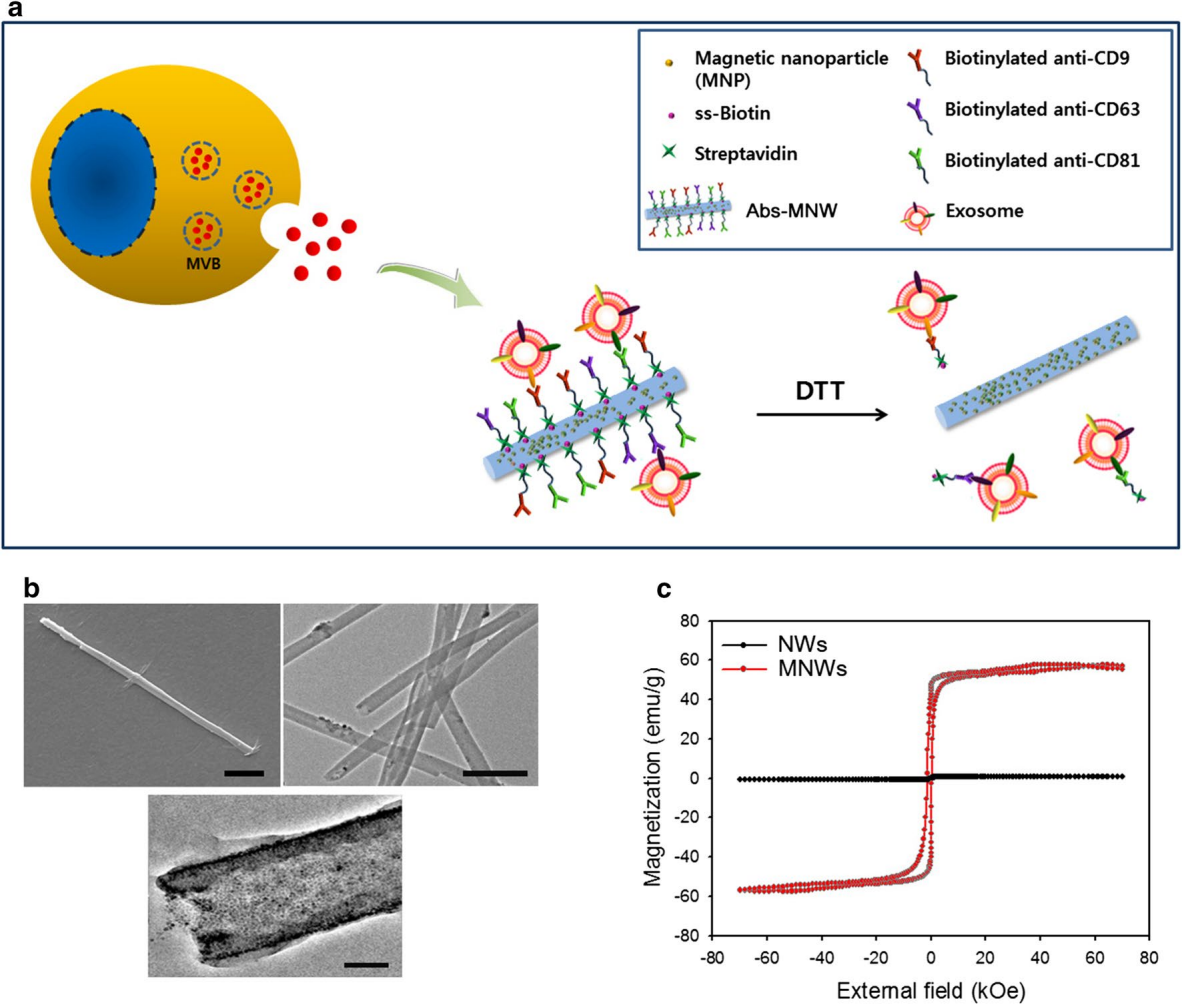
**a** An illustration showing the antibody cocktail-conjugated magnetic nanowires (Abs_MNWs) used for the isolation of circulating exosomes. **b** Scanning electron microscopy (left: scale bar, 500 nm) image and transmission electron microscopy (right: scale bar, 500 nm and bottom: scale bar, 100 nm) image of Abs_MNWs. **c** Magnetic hysteresis loop of magnetic nanowires (MNWs) and bare nanowires (NWs) at room temperature

Circulating tumor-related biomarkers (circulating tumor cells [CTCs], cell-free DNA [cfDNA], exosomes, etc.) have been recognized as a valuable evidence for real-time non-invasive assessment of cancer prognosis and response to therapy [1]. Although CTCs and cfDNAs have provided great insight into cancer progression, identification and enumeration of these rare markers are technically challenging, owing to their extreme rarity in bloodstream. On the other hand, considerable attention has been focused on the isolation and detection of circulating tumor-derived exosomes. As most eukaryotic cells shed extracellular vesicles (EVs), there is a need for the development of a well-defined highly advanced technique, which may allow enhanced recovery of exosomes from biological samples. These exosomes may help researchers to elucidate and support the quantitative and qualitative aspects of the complex tumor dynamics [2–6]. Unlike the extracellular microvesicles (EMVs; 500–5000 nm diameter) that are secreted directly from the cell membrane, exosomes (30–150 nm diameter) originate from the endolysosomal pathway and possess key molecular components from the cell of origin. In particular, tumor cell-derived exosomes have been reported to facilitate, at molecular level, the progression, invasion, and metastasis of cancer cells that are subsequently implicated in modulating tumor pathogenesis and progression [7–9]. Hence, the ability to detect and isolate tumor-derived exosomes may facilitate researchers to explore intracellular signals between cells and analyze functional molecular components (proteins, mRNA, and microRNA), which may provide crucial information about cancer diagnosis and prognosis. Current exosome isolation techniques include ultracentrifugation, density gradient centrifugation, size exclusion chromatography, exosome precipitation, and immunoaffinity capture, whereas characterization methods include western blotting and ELISA [10–14]. Although these methods are widely used to purify and analyze exosomes, their translation into clinical applications is often impractical due to the shortcomings of the existing technology. For instance, the ultracentrifugation method, regarded as the gold standard for exosome isolation, is labor-intensive and time-consuming and requires a large amount of sample as well as costly specialized equipment. This results in relatively low efficiency and purity of the isolated exosome. Therefore, the development of a technically simple and ultrasensitive technique would be beneficial for the isolation and molecular analysis of circulating exosomes in diverse body fluids such as blood, urine, saliva, semen, and ascites, even in a small amount of sample. Accordingly, it is important to establish more affordable and accessible platforms that exhibit great sensitivity, high throughput, and relatively low cost, which may improve cancer treatment outcomes. Our recent study demonstrated a novel strategy for the recovery and detection of CTCs and cfDNA from blood or urine samples of cancer patients using multifunctional nanostructures by readily regulating topographical, electrical and chemical cues of the nanoscale substrates [15–18]. As an extended study, here we developed an immuno-magnetic strategy for efficient and simple isolation of exosomes. Elongated magnetic nanowires (MNWs) doped with a large amount of magnetic nanoparticles (MNPs) and biotin moieties are capable of conjugating with diverse exosome-specific antibodies such as anti-CD9, anti-CD63, and anti-CD81 via streptavidin–biotin interaction (Fig. 1). We used this approach for efficient extraction and quantification of exosomes without the need for expensive instruments and complex sample preparation steps, within 1 h. Owing to their small lateral size, elongated structure, high surface-to-volume ratio, and strong magnetism, nanowires are improved approach for the elution of exosomes with reliability, reproducibility, and convenience, with potential applications in routine clinical workflow.

## Results and discussion

### Preparation and characterization of antibody cocktail-conjugated magnetic nanowires (Abs_MNWs)

We have recently demonstrated MNWs as a highly efficient platform for the capture and enrichment of CTCs and cfDNA from biological samples (blood or urine) of cancer patients [15]. The nanowire-based approach can greatly improve the recovery yield and purity by specifically enhancing the interaction with tumor-specific biomarkers in biologically complex fluids. The enhanced interaction is most likely attributed to the following inherent topographic features of nanowire: (i) a large surface area that allows direct incorporation of, or modification with, available functional groups; (ii) long and thin morphology that assists in detection and capture of tumor-associated markers, with minimum steric hindrance between the nanostructure and other components present in the blood or urine; and (iii) the elongated structure that is capable of encapsulating large amounts of iron oxide nanoparticles (~ 10 nm) in its interior during electrochemical deposition, which has significant impact on the magnetic response of the resulting nanowires.

Circulating tumor-derived exosomes are known to play a key role in the process of carcinogenesis. As exosomes are released at a high level during cancer progression, exosome amount detected from cancer patients is much higher than that from healthy individuals. Thus, exosomes can serve as a valuable biomarker with significant clinical relevance in both biological and clinical research. Despite tremendous progress in the exosome extraction techniques, there is a need for standard optimized protocols. We applied the nanowire-based strategy for the isolation and purification of exosomes from the plasma of cancer patients. The SS-biotin- and MNP-doped Ppy nanowires were electrochemically deposited within the well-ordered nanoporous AAO template using a mixture of pyrrole monomers, MNPs, and SS-biotin. After removing the AAO template completely, the resulting MNWs were further labeled with a cocktail of antibodies that have been considered as a versatile and efficient platform for exosome capture as well as DTT-mediated release of the captured exosome with ease, robustness, and efficiency (Fig. 1a; Abs_MNWs). Thin elongated Abs_MNWs are capable of providing sufficient binding sites to covalently link antibodies specific to exosomes (anti-CD9, anti-CD63, and anti-CD81) via biotin-streptavidin interactions. As members of the tetraspanin family of proteins, CD9, CD63, and CD81 are overexpressed in exosomes, predominantly located on the surface, thereby serving as a potential exosomal marker. Abs_MNWs may offer several advantages in the extraction and identification of exosomes with phenotypic variation while reducing the inevitable loss of circulating exosomes during the capture process. MNWs displayed an average length of 18 μm and diameter of 200 nm, as observed by SEM and TEM (Fig. 1b; left and middle). The assemblies of randomly distributed, highly packed MNPs embedded in Abs_MNWs were revealed by TEM image (Fig. 1b; right). As a result of the high density of MNPs, MNWs can possess a high saturation magnetization (Ms = 57 emu/g), while no magnetic response was observed with bare nanowires (Fig. 1c).

### Analysis of exosomes isolated from cell lines by magnetic beads and magnetic nanowires

**Fig. 2:**
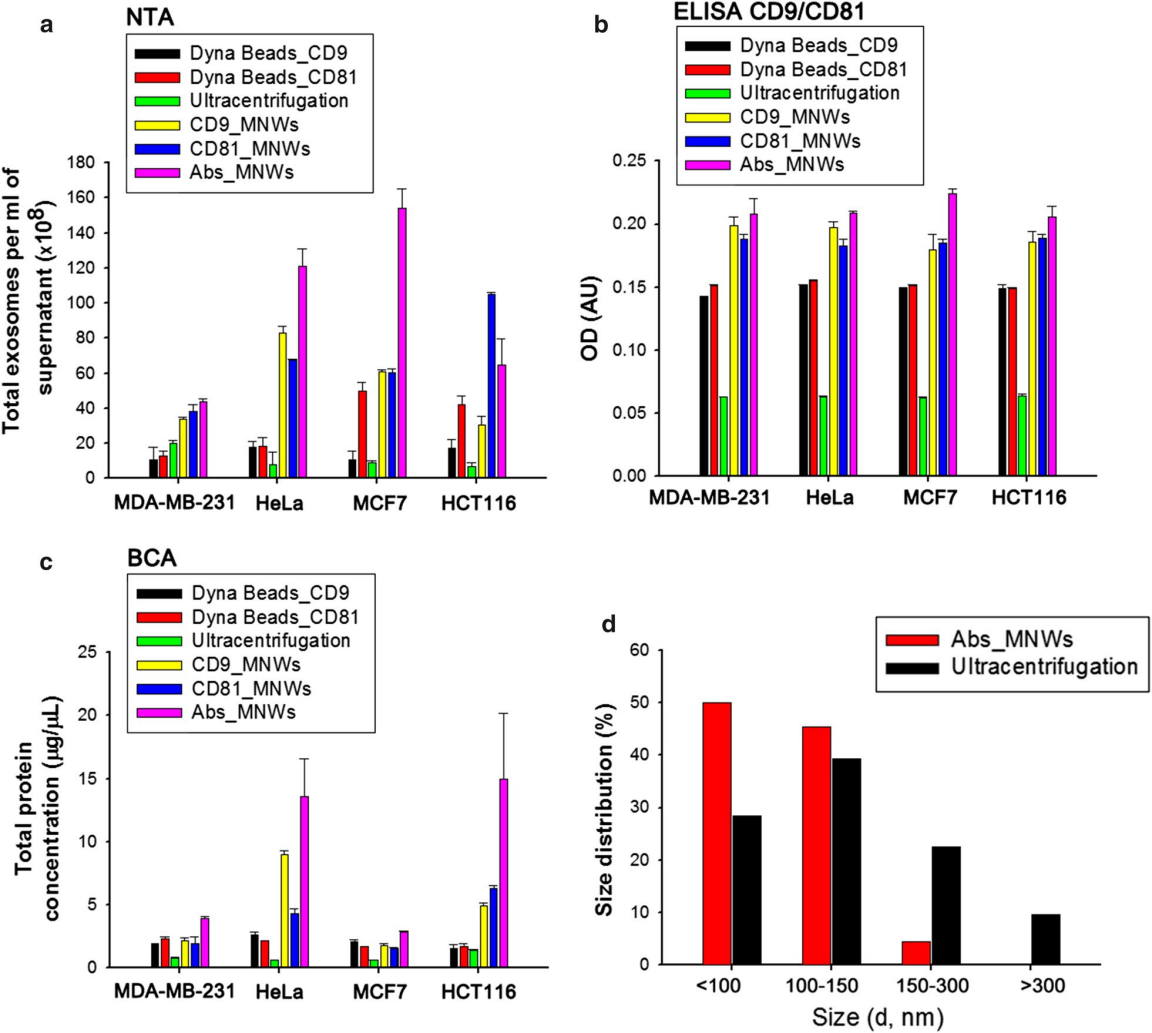
**a** NTA analysis of exosomes isolated from MDA-MB-231, HeLa, HCT116, and MCF7 cells by different recovery methods (Dyna Beads_CD9, Dyna Beads_CD81, CD9_MNWs, CD81_MNWs, and Abs_MNWs), where the amount of antibodies directly conjugated to Dyna Beads or MNWs is equivalent to 200 pg/mL. **b** ELISA results indicating that exosomes recovered from Abs_MNWs showed higher levels of CD9/CD81-specific exosomes as compared with Dyna Beads. **c** Concentration of total proteins in exosomes isolated from MDA-MB-231, HeLa, HCT116, and MCF7 cells by the other recovery methods. **d** Representative size distribution of exosomes isolated using Abs_MNWs (manually determined from electron micrographs)

As a proof-of-concept study, we investigated the nanowire-based approach for the isolation of exosomes from concentrated culture medium (CCM) while minimizing the non-specifically bound protein aggregates and membrane vesicles (Fig. 2). First, we evaluated the performance of Abs_MNWs using four different cancer cell lines. These included MDA-MB-231 and MCF7 breast cancer cells, HCT116 colon cancer cells, and HeLa cervical cancer cells. The efficacy of exosome recovery with magnetic beads conjugated with anti-CD81 (Dyna Beads^®^_CD81) and anti-CD9 (Dyna Beads^®^_CD9) was compared with that of recovery by magnetic nanowires conjugated with anti-CD9 (CD9_MNWs) and anti-CD81 (CD81_MNWs). Exosomes isolated by the five different methods were validated by nanoparticle tracking analysis (NTA), ELISA, and protein concentration (bicinchoninic acid assay, BCA) (Fig. 2a–c). For all cell types, the Abs_MNWs treatment resulted in a high yield and purity of isolated exosomes. In particular, CD9/CD81 sandwich ELISA results exhibited the highest optical density (OD) for exosomes retrieved by Abs_MNWs in comparison with those for samples obtained using the other methods. Abs_MNWs with all three different types of antibodies (CD9, CD81, and CD63) showed significantly greater amounts of bound exosomes compared with the magnetic beads and magnetic nanowires conjugated with single CD81 or CD9 antibody. The use of three types of exosome-specific antibodies, rather than a single antibody, proved to be a more sensitive and specific strategy in exosome extraction and protein analysis. The results indicated that the nanowire-based approach offers an accessible, versatile, and flexible method for exosome isolation, with a short recovery time of less than 1 h and significantly improved efficiency. The diameter of exosomes isolated by Abs_MNWs was mostly in the range of 40–150 nm, indicative of the homogenous size distribution of exosomes isolated using nanowires (Fig. 2d).

### Evaluation of exosomes collected from the plasma of healthy donors and patients with breast and lung cancer using Abs_MNWs

**Fig. 3:**
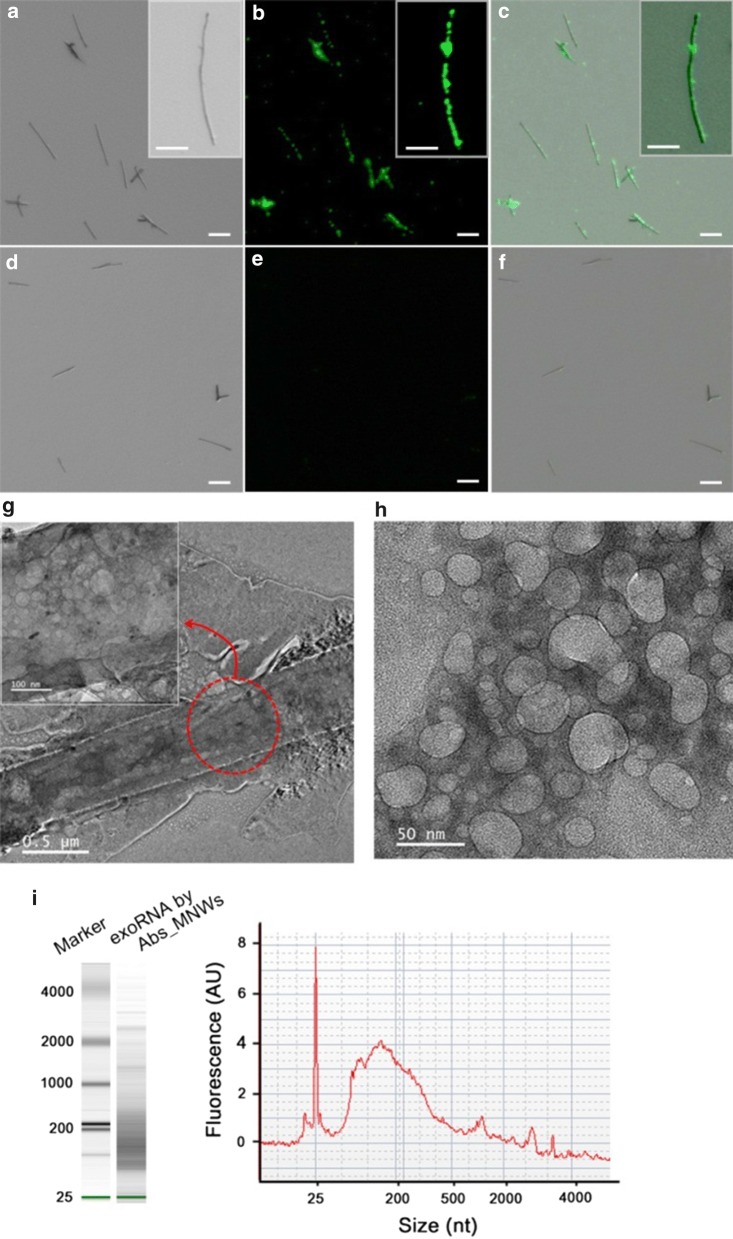
**a**–**c** Fluorescence images of exosomes captured on Abs_MNWs from the plasma of lung cancer patients. **d**–**f** Fluorescence images of exosomes that were immediately released from the Abs_MNWs after incubation with DTT for 30 min. Exosomes captured and released by the magnetic nanowires were detected under the fluorescence microscope following staining with DiO dye. All fluorescence images were acquired under same condition (scale bar, 10 µm; inset scale bar, 5 µm). **g** TEM images showing exosomes captured on Abs_MNWs from the plasma of lung cancer patients (scale bar, 500 nm; inset scale bar, 100 nm). Inset exhibits an image at higher magnification. **h** Representative TEM images showing exosomes released from the plasma of lung cancer patients using Abs_MNWs (scale bar, 50 nm). **i** Bioanalyzer size distribution of exosomal RNA that were extracted from exosomes isolated by Abs_MNWs

We explored the ability of Abs_MNWs to recover exosomes from the plasma of lung cancer patients (Fig. 3a–f). Exosomes captured on the nanowires were labeled with membrane-specific fluorescence dye DiO (Fig. 3a–c).

**Fig. 4:**
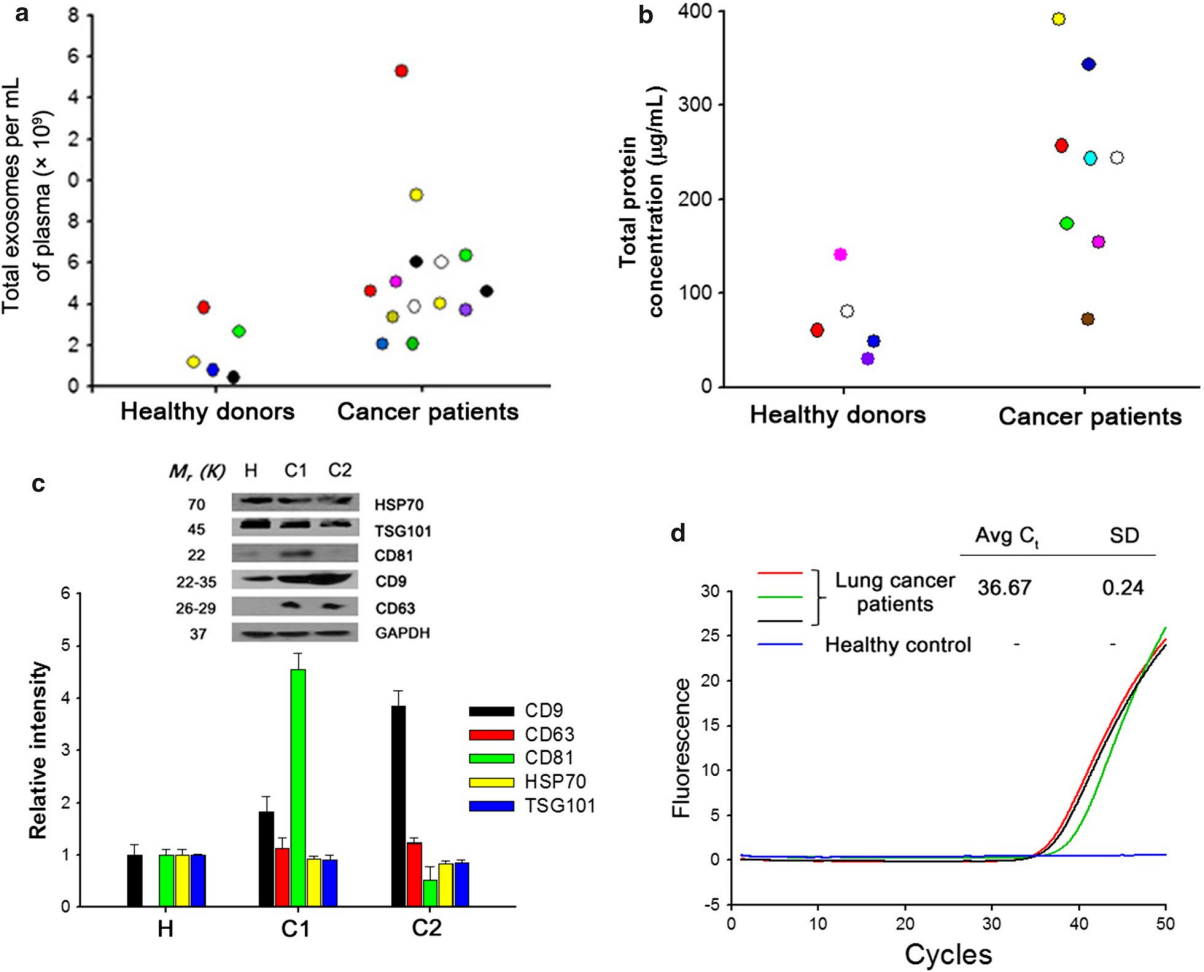
**a** NTA results showing the number of exosomes collected from the plasma of healthy donors and patients with breast and lung cancer using Abs_MNWs. **b** Quantification of total proteins in exosomes isolated using Abs_MNWs from the plasma of healthy donors and patients with breast and lung cancer. **c** Western blotting with antibodies against HSP70, TSG101, CD81, CD9, CD63, and glyceraldehyde-3-phosphate dehydrogenase (GAPDH) for exosomes isolated using Abs_MNWs from the plasma of healthy donors (H) and lung cancer patients (C1, C2). **d** RT-PCR result demonstrating the expression levels of miR-21 in exosomes after extraction from the plasma of healthy controls and lung cancer patients by Abs_MNWs

Strong fluorescent signals were obtained from the surface of the nanowire, confirming its direct attachment to exosomes. No fluorescent signal was detected from the nanowires treated with DTT, indicative of the DTT-mediated release of exosomes for their complete recovery from the nanowires (Fig. 3d–f). TEM analysis revealed diverse variations in exosome morphology, showing roughly spheroidal vesicles with diameters of 40–150 nm (Fig. 3g–h). We extracted RNA from exosomes and examined the Bioanalyzer profile of exosomal RNAs to assess their integrity, purity, and size distribution (Fig. 3i). A wide range of RNA sizes (mostly less than 400 nucleotides) were obtained with Abs_MNWs, and majority of these displayed a size of approximately 170 nucleotides in the electropherogram. Furthermore, we measured the total exosome levels in plasma of healthy donors and cancer patients by analyzing exosomes captured onto Abs_MNWs (Fig. 4a). In comparison to the healthy controls, cancer patients showed a threefold increase in the secretion of circulating exosomes. These results are in line with those previously reported, wherein elevated levels of exosomes were observed in body fluids of cancer patients. Moreover, cancer patients showed a 3.9-fold increase in exosomal protein levels as compared with the healthy controls, as revealed by the bicinchoninic acid assay (Fig. 4b).

**Fig. 5:**
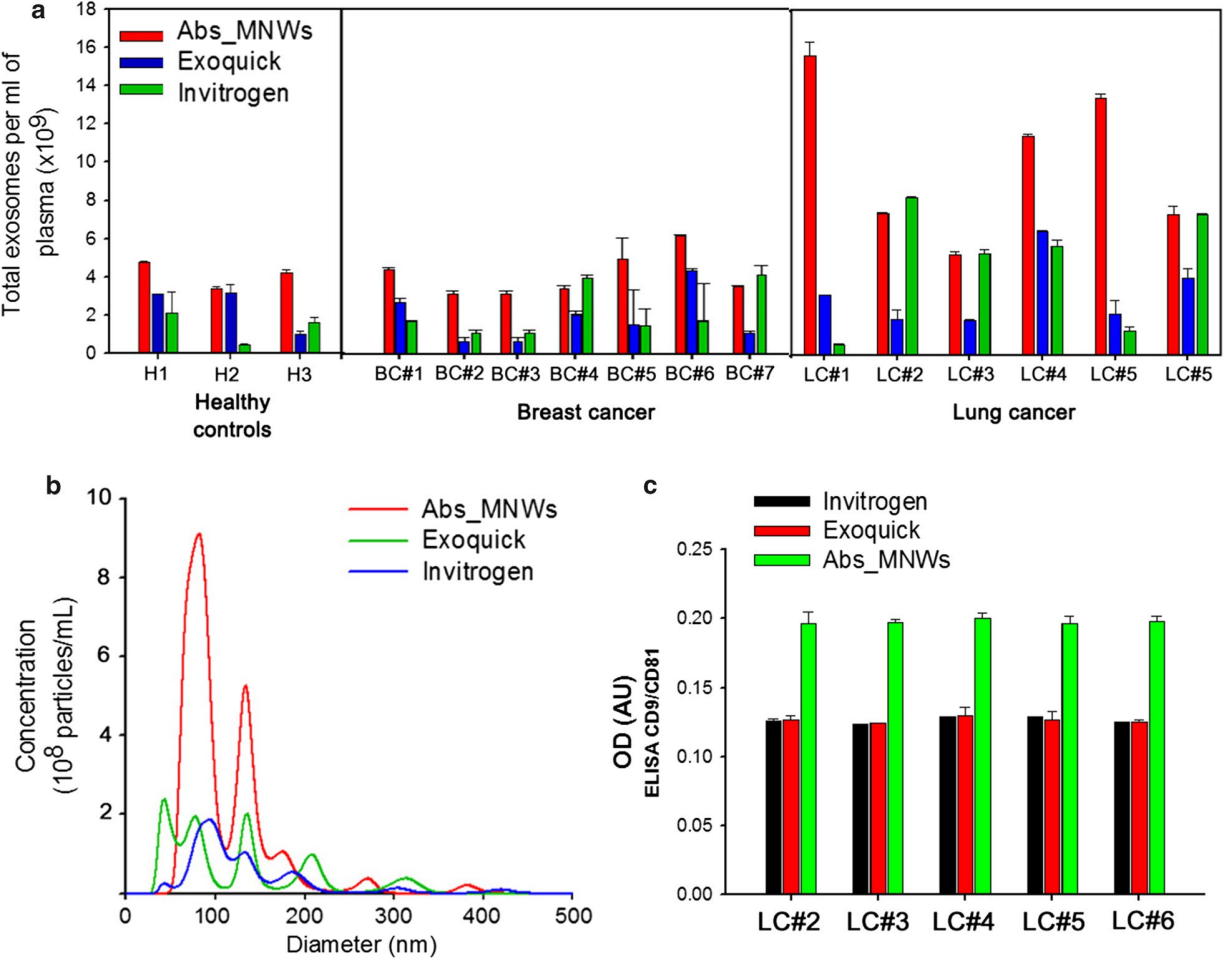
**a** Comparison between Abs_MNWs and conventional methods (Exoquick and Invitrogen) for exosome isolation. **b** NTA results demonstrating the ability of nanowires to isolate exosomes from the plasma of lung cancer patients versus that of the Exoquick and Invitrogen methods. **c** Comparison of exosome sandwich ELISA. The exosomes were isolated from lung cancer patients using Abs_MNWs and conventional methods (Exoquick and Invitrogen). The Abs_MNWs showed excellent performance in retrieving exosomes, particularly with CD9/CD81 exosomal specific proteins. The data shown represent the mean ± SD of data from three independent experiments

Efficient isolation of exosomes by Abs_MNWs was validated through the quantitative analysis of common exosomal markers, including CD9, CD81, CD63, TSG101, and HSP70 [19, 20]. Aside from the confirmation of their shape and size, our results revealed that the vesicles isolated with Abs_MNWs contained various exosomal proteins and, thus, were deemed as genuine exosomes (Fig. 4c). The exosomal RNAs were further amplified using a cDNA synthesis kit and evaluated for the expression of miR-21, given the biological and clinical significance of miRNAs [21, 22]. Evaluation of the expression levels of exosomal miRNA after extraction from the plasma of healthy controls and lung cancer patients by Abs_MNWs indicated that distinct exosomal miR-21 signatures were observed in lung cancer patients (Fig. 4d). We compared the yield, size distribution, and amount of total proteins in exosomes isolated from healthy subjects and cancer patients using the three different methods. As shown in Fig. 5a, Abs_MNWs achieved a higher yield and purity of exosomes isolated from the plasma of cancer patients, with an average NTA value of 6.3 ± 0.15 × 10^9^ particles/mL. On the other hand, the concentration of exosomes isolated from the plasma of cancer patients using Exoquick and Invitrogen kits was 2.4 ± 0.12 × 10^9^ and 1.73 ± 0.26 × 10^9^ particles/mL, respectively.

Thus, Abs_MNWs showed about threefold higher yield as compared to the two conventional methods. In addition, the size distribution of majority exosomes isolated by Abs_MNWs was uniform and in the range of 40–150 nm (Fig. 5b). Higher levels of exosomal proteins were identified with Abs_MNWs versus other two methods (Fig. 5c). In this study, we described a simple, rapid, and sensitive method of isolating exosomes from small sample volumes using Abs_MNWs. The procedure, processing time, cost, and minimum sample volume required for the cfDNA extraction by Abs_MNWs are summarized in Table 1.

**Table 1:** Summary of the characteristics of the Abs_MNWs method for exosome isolation

	Abs_MNWs
Procedure	
	(1) Exosome capture by Abs_MNWs, 30 min (2) Place the tube (1) in a magnetic separation rack and wash with PBS, 5 min (3) Elute exosomes from Abs_MNWs by incubating in DTT solution, 25 min
Processing time	60 min
Cost (per 1 mL sample)	11$
Minimum volume of plasma	250 μL

Thus, nanowire-based method allows isolation of homogeneous population of exosomes with higher yield and purity and displays potential applications for the analysis of protein, lipid, mRNA, and miRNA from highly purified exosomes. This may be useful in studying the biological functions and role of exosomes in cancer development.

## Conclusions

We demonstrate a novel approach for efficient isolation and detection of exosomes using Abs-MNWs coupled to different types of exosome-specific antibodies and high density of MNPs. The elongated morphology of nanowires affords more flexibility and versatility for exosome isolation and identification by facilitating multiple interactions through recognition receptors on exosomes, thereby resulting in enhanced exosome recovery even from small volumes of blood plasma of cancer patients. Overall, the simplicity of preparation and excellent performance of the nanowire-based strategy offer high sensitivity and specificity in exosome isolation and detection, which can be widely applied to a variety of cancer types for cancer screening and diagnosis.

## Methods

### Chemicals and reagents

Pyrrole, poly(sodium 4-styrenesulfonate) (PSS), *N*-(3-dimethylaminopropyl)-*N*′-ethylcarbodiimide hydrochloride (EDC), *N*-hydroxysuccinimide (NHS), iron oxide (II, III), MNP solution (average diameter, 10 nm), streptavidin, and sodium hydroxide (NaOH) were obtained from Sigma Aldrich (St. Louis, MO, USA). An anodized aluminum oxide (AAO) membrane filter (pore diameter, 200 nm) was purchased from Whatman (Pittsburgh, PA, USA). NHS-SS-biotin was supplied by CovaChem (Loves Park, Illinois, USA). Biotinylated anti-CD63 and anti-CD81 were obtained from AnCell (Oak Park, Minnesota, USA). Biotinylated anti-CD9 was procured from Abcam (Cambridge, UK). Anti-CD9, anti-CD63, anti-CD81 was purchased from Cell Signaling Technology (Denver, MA, USA). Invitrogen™ Exosome-Human CD9 Isolation Reagent from cell culture (Dyna Beads_CD9) and Invitrogen™ Exosome-Human CD81 Isolation Reagent from cell culture (Dyna Beads_CD81) were obtained from ThermoFisher Scientific Inc. (Waltham, MA, USA).

### Fabrication and characterization of anti-CD9-conjugated, anti-CD81-conjugated, and antibody cocktail-conjugated magnetic nanowires (CD9_MNWs, CD81_MNWs, and Abs_MNWs)

We prepared CD9_MNWs, CD81_MNWs, and Abs_MNWs as previously described [23]. Briefly, MNPs (average diameter, 10 nm) were incubated inside the pore of Au-coated AAO membrane filter (pore diameter, 200 nm), followed by gentle aspiration. Electrochemical experiments were conducted using a potentiostat/galvanostat (BioLogic SP-50), with a Pt wire, Ag/AgCl reference, and Au-coated AAO membrane used as a counter, reference, and working electrode, respectively. Polypyrrole (Ppy) was electrochemically polymerized to the pores of AAO membrane in a solution containing 0.1 M pyrrole, 0.01 M PSS, and 1 mM NHS-SS-biotin by applying chronoamperometry (CA) at 1.5 V for 7 min. The resulting AAO membranes were washed several times with distilled water and incubated in 2 M NaOH for 2 h to remove AAO template. In the subsequent steps, 6 mM NHS and 30 mM EDC were added to the resulting MNWs and incubated for an additional 45 min. MNWs were immersed in streptavidin (10 µg/mL) for 45 min at room temperature, followed by washing with water. After streptavidin labeling, the biotinylated antibody cocktail (i.e., biotinylated anti-CD9, biotinylated anti-CD63, and biotinylated anti-CD81 in Dulbecco’s phosphate-buffered saline) was conjugated to streptavidin-labeled MNWs at 4 °C overnight to obtain the final product (i.e., Abs_MNW) with a final antibody concentration of 0.4 µg/mL. For the preparation of anti-CD81 or anti-CD9 conjugated magnetic nanowires, biotinylated anti-CD81 or biotinylated anti-CD9 was linked to streptavidin-labeled MNWs at 4 °C overnight to produce the final product (i.e., CD81_MNWs or CD9_MNWs) with a final antibody concentration of 0.4 µg/mL. The mean number of antibodies bound per MNW was determined using a previously described assay [24]. Briefly, we detected and quantified the amount of antibodies conjugated onto 1.26 × 10^6^ MNWs/mL by incubating them with horseradish peroxidase-labeled (HRP) anti-mouse IgG for 1 h, where a 3% solution of bovine serum albumin (BSA) was employed to prevent nonspecific binding. MNWs were washed carefully several times to remove any unbound IgG. HRP attached to the MNWs was further treated with 3,3′,5,5′-tetramethyl benzidine, producing a colored product; the relative amount of antibody was determined in comparison with an HRP anti-mouse IgG standard curve. The results were read using a spectrophotometer at 650 nm. The morphology of Abs_MNW was observed by scanning electron microscopy (SEM; JSM-6701F, JEOL) with an accelerating voltage of 15 kV and transmission electron microscopy (TEM; G2F30, Tecnai) with an accelerating voltage of 300 kV. Magnetic measurements were carried out using a SQUID-VSM magnetometer (MPMS-VSM, Quantum Design, San Diego, CA, USA) with the applied magnetic field in the range from 70 to − 70 kOe.

### Cell culture and preparation of concentrated culture medium (CCM)

Four different types of cancer cell lines (MDA-MB-231 and MCF7 breast cancer cells, HCT116 colon cancer cells, and HeLa cervical cancer cells) were cultured in Roswell Park Memorial Institute (RPMI)-1640 medium (Invitrogen, Carlsbad, CA) containing 10% fetal bovine serum (FBS) and 1% penicillin–streptomycin at 37 °C in a 5% CO_2_ atmosphere. The cells (~ 2 × 10^9^ cells) were pelleted and washed thrice with RPMI-1640 media, followed by replacement of media with serum-free RPMI media. The cells were cultured for additional 2 days in serum-free RPMI media before exosome harvesting. Intact cells and cell debris were removed by centrifugation at 300×*g* for 10 min and 2000×*g* for 20 min, respectively. CCM was collected and filtered through sterile 0.22-µm (pore-size) syringe filter (Merck Millipore, USA) [2, 4, 14, 25].

### Exosome isolation by Dyna Beads_CD9, Dyna Beads_CD81, CD9_MNWs, CD81_MNWs, and Abs_MNWs

For isolation of circulating exosomes, Dyna Beads_CD9 (5.0 × 10^5^ Beads/µL), Dyna Beads_CD81 (5.0 × 10^5^ Beads/µL), CD9_MNWs (1.0 × 10^3^ MNWs/µL), CD81_MNWs (1.0 × 10^3^ MNWs/µL), and Abs_MNWs (1.0 × 10^3^ MNWs/µL) were incubated in 250 µL–3 mL CCM or plasma of healthy donors and cancer patients for 30 min at room temperature with gentle shaking to promote attachment of exosomes. Next, a magnetic field created by the MagneSphere^®^ Technology Magnetic Separation Stands (Promega, USA) was applied on the sample tubes (1.5 mL microcentrifuge tubes) to efficiently remove the supernatant and collect the captured exosomes. Dithiothreitol (DTT) solution (50 mM) was added to the resulting solution to release the captured exosomes from the nanowires by breaking disulfide bonds. We evaluated the concentration and size of exosomes isolated by MNWs using the nanoparticle tracking analysis (NTA; NanoSight NS300, Malvern Instruments, Malvern, UK) and Malvern Zetasizer Nano-Z (Malvern Instruments, Malvern, UK). In addition, total protein concentration was determined using the bicinchoninic acid (BCA) assay kit (Thermo Scientific, Waltham, MA, USA) according to the manufacturer’s instructions. Briefly, 1 µL of isolated exosome was diluted in 19 µL of M-PER reagent (Thermo Fisher Scientific, Massachusetts, Waltham, USA) and 200 µL of BCA reagent A and B mixture (A:B = 50:1) was added and incubated for 30 min at 37 °C. The optical density (OD) of the sample was measured by a UV/VIS spectrophotometer at a wavelength of 562 nm. The protein concentration was calculated from standard BCA curve (r^2^ = 99.8%). All measurements were carried out under constant experimental conditions to obtain comparable results. For exosome sandwich ELISA assay, 100 µL of anti-CD9 antibody (1 µg/100 µL) was coated onto 96 well plate (Thermo Fischer Scientific) and incubated at 4 °C overnight. Then, the plate was blocked with 1% BSA in PBS buffer at 37 °C for 1 h. After washing with 0.1% BSA-PBS buffer three times, the plate was incubated with an exosome solution in PBS buffer (100 μL) at 37 °C for 1 h. Upon removing the solution, the plate was washed twice with 0.1% BSA-PBS buffer and added to biotin-conjugated detection antibodies (anti-CD81; LifeSpan Biosciences, Inc., Seattle, WA, USA) in PBS buffer (100 μL; 500 ng/mL), followed by incubating at room temperature for 1 h. After washing three times with 0.1% BSA-PBS buffer, the plate was incubated again with a solution of HRP conjugated streptavidin in PBS buffer (100 μL; 1:1000) at room temperature for 30 min and then washed three times with 0.1% BSA-PBS buffer. TMB Ready Solution (Thermo Fisher Scientific) was then added to the plate and incubated at room temperature for 15 min, followed by the addition of 50 μL of stop solution to each well. The absorbance was read using a UV/VIS spectrophotometer at a wavelength of 450 nm.

### Exosome isolation by commercial extraction kits

Exosomes were isolated and purified using ExoQuick (EXOQ5TM-1, System Biosciences, Palo Alto, CA, USA), Invitrogen Total Exosome Isolation Kit (4484451, Thermo Fisher Scientific, Massachusetts, Waltham, USA), and Exosome-Human CD81 Flow Detection Reagent (10622D, Thermo Fisher Scientific, USA) according to the manufacturer’s instructions. Briefly, the reagents were added to CCM or plasma of healthy donors and cancer patients to isolate exosomes and the mixture was vortexed and centrifuged at 4 °C as described in the manufacturers’ protocols. The pellet containing exosomes was resuspended in DPBS or ultrapure water. Subsequently, the exosome pellet was diluted in M-PER reagent (Thermo Fisher Scientific, Massachusetts, Waltham, USA) and BCA reagent A and B (A:B = 50:1) was added and incubated for 30 min at 37 °C. The protein concentration of the pellet was determined using the BCA protein assay kit (Thermo Scientific, Waltham, MA, USA) according to the manufacturer’s instruction. To improve reproducibility, all assays were carried out under same experimental conditions.

### Blood specimen collection and preparation

Whole blood was collected in Vacutainer tubes containing the anti-coagulant ethylenediaminetetraacetic acid (EDTA), following procedures approved by the National Cancer Center Institutional Review Board. The collected blood was centrifuged at 3000×*g* for 10 min for separation of plasma, which was stored at − 80 °C until analysis.

### Transmission electron microscopy (TEM) analysis of exosomes

Freshly isolated exosomes from cells were resuspended in cold DPBS. Exosome samples were prepared for TEM analysis using exosome-TEM-easy kit (101Bio, Palo Alto, CA, USA) according to the manufacturer’s instruction. Briefly, re-suspended exosomes were mounted on Formvar-carbon coated EM mesh 400 grids and incubated for 10 min. The resulting grids were rinsed twice with wash buffer and deposited on the EM solution for 10 min. After washing and dehydration steps, exosomes were subjected to TEM with an accelerating voltage of 300 kV.

### Fluorescence analysis of exosomes

The captured and released exosomes on/from the Abs_MNWs were labeled with a fluorescent Vybrant™ DiO dye solution (5 µL/mL, Molecular Probes, Life Technologies) by incubating for 8 min at 37 °C to allow staining of the exosomal membrane. The exosomes were rinsed with phosphate-buffered saline (PBS) and the DiO-labeled exosomes were analyzed by a Zeiss fluorescence microscope.

### Western blotting

Exosomes isolated by Abs_MNWs were lysed in M-PER reagent (Thermo Fisher Scientific, Massachusetts, Waltham, USA). Protein samples (20 µg) were separated on a 10% sodium dodecyl sulfate polyacrylamide gel and transferred onto polyvinylidene difluoride (PVDF) membranes (0.45 µm, Millipore). The membranes were blocked with 3% skim milk for 1 h at room temperature and probed with primary mouse anti-TSG101 (1:1000), rabbit anti-HSP70 (1:1000), rabbit anti-glyceraldehyde 3-phosphate dehydrogenase (1:1000), rabbit anti-CD9 (1:1000), rabbit anti-CD63 (1:1000), rabbit anti-CD81 (1:1000), and rabbit monoclonal anti-GAPDH (1:1000) for overnight. Following incubation, the membranes were incubated with an appropriate secondary antibody (goat anti-mouse IgG [1:3000] or goat anti-rabbit IgG [1:3000]) for 1 h. Blots were washed three times with TBST buffer after each incubation step and visualized using a SuperSignal^®^ West Pico Chemiluminescent Substrate reagent (34077, Thermo Scientific).

### RNA extraction and miRNA analysis

Total exosomal RNA was extracted with TRIzol (Invitrogen, Paisley, UK) and homogenized through a pipette following the manufacturer’s protocol. Further, RNA samples were treated with chloroform (Merck, Darmstadt, Germany) and centrifuged for 15 min at 12,000×*g* at 4 °C to separate the mixture into aqueous and organic phases, and isopropanol was used to precipitate the supernatant. Then, miR21-specific complementary DNA (cDNA) was synthesized from 10 ng of RNA eluate with random hexamers using TaqMan MicroRNA reverse transcription Kit (Applied Biosystems, Foster city, CA, USA). The quantitative reverse transcription polymerase chain reaction (qRT-PCR) was performed using an LC480 real-time PCR system (Roche, Basel, Switzerland) for 5 min at 25 °C, 20 min at 46 °C, and 1 min at 95 °C, followed by storage at 4 °C. We used pre-designed primers and probe (TaqMan™ Advanced miRNA Assay, Thermo Fisher Scientific) for miR-21 (has-miR-21-3p, assay ID 477973_mir), prepared according to the manufacturer’s instructions.

## Abbreviations

Ppy: polypyrrole
NWs: nanowires
Abs_MNWs: the magnetic nanowires conjugated with a large amount of magnetic nanoparticles and antibody cocktails
CTCs: circulating tumor cells
cfDNA: cell-free DNA
EVs: extracellular vesicles
EMVs: extracellular microvesicles
MNWs: magnetic nanowires
MNPs: magnetic nanoparticles
PSS: poly(sodium 4-styrenesulfonate)
EDC: *N*-(3-dimethylaminopropyl)-*N*′-ethylcarbodiimide hydrochloride
NHS: *N*-hydroxysuccinimide
AAO: anodized aluminum oxide
CA: chronoamperometry
SEM: scanning electron microscopy
TEM: transmission electron microscopy
CCM: concentrated culture medium
DTT: dithiothreitol
NTA: nanoparticle tracking analysis
qRT-PCR: quantitative reverse transcription polymerase chain reaction
